# Full-endoscopic spine-surgery in the elderly and patients with comorbidities

**DOI:** 10.1038/s41598-024-80235-2

**Published:** 2024-11-25

**Authors:** Jannik Leyendecker, Tobias Prasse, Pia Rückels, Malin Köster, Lena Rumswinkel, Valentina Schunk, Isabella Marossa, Peer Eysel, Jan Bredow, Christoph P. Hofstetter, Imad Khan

**Affiliations:** 1 Department of Orthopedics and Trauma Surgery, Faculty of Medicine and University Hospital Cologne, University of Cologne, Cologne, Germany; 2 Department of Neurological Surgery, University of Washington, 325 9th Avenue, Box 359924, Seattle, WA 98104 USA; 3 Department of Orthopedics and Trauma Surgery, Krankenhaus Porz am Rhein, University of Cologne, Cologne, Germany

**Keywords:** Orthopaedics, Risk factors

## Abstract

Due to demographic changes, a growing number of elderly patients with comorbidities will require spine surgery in the next decades. However, age and multimorbidity have been associated with considerably worse postoperative outcomes, and is often associated with surgical invasiveness. Full-endoscopic spine-surgery (FESS), as a cornerstone of contemporary minimally invasive surgery, has the potential to mitigate some of these disparities. Thus, we conducted an analysis of all FESS cases at a national center. Utilizing the Charlson Comorbidity index (CCI) ≥ 3 as a frailty surrogate we separated patients in two groups for patients with and without comorbidities. Patients with (CCI) ≥ 3 exhibited a higher age (*p* < 0.001), and number of comorbidities (*p* < 0.001) than the control group. Thereafter, a propensity score matching was done to adjust for potential confounders. Postoperative safety measures in emergency department utilization, and clinic readmission did not significantly differ between the groups. Furthermore, patients of both groups reported similar postoperative pain improvements. However, patients with a (CCI) ≥ 3 were treated as inpatients more often (*p* < 0.001), had a higher length of stay (*p* < 0.001) and a smaller functional improvement after at a chronic postoperative timepoint (*p* = 0.045). The results underline safety and efficacy of FESS in patients with comorbidities. Additionally, they provide guidance for preoperative patient counselling and resource utilization when applying FESS in frail patients.

## Introduction

The growing number of degenerative spine diseases, due to demographic ageing, will be the leading cause of several challenges for global healthcare systems, in the coming decades. Due to an emerging number of indications to address pathologies in the aging spine, limited hospital resources run the risk of getting overwhelmed by a rising number of spine surgeries^1^. Frail and elderly patients are especially susceptible to adverse outcomes following elective spine surgery due to destabilizing stressors and a reduced ability to regulate homeostasis^2^. Various frailty assessments and surrogates have shown a correlation between patient age, comorbidity risk and frailty status^3,4^. One such frailty surrogatesis the Charlson Comorbidity Index (CCI), which is recognized by clinicians as an accurate predictor of spinal surgical outcomes in susceptible patients^5–7^. As opposed to other frailty surrogates (e.g. MFI), the CCI includes age as an item which has substantial relevance for preoperative patient counseling and selection. Frailty is associated with a multitude of adverse outcomes and quality surrogates after spine surgery including mortality, surgical complications, reoperation, wound complications, non-home discharge, length of stay, and hospital readmissions^8–13^. Such negative post-operative complications occur at a higher likelihood when paired with considerable surgical invasiveness as well as advanced age^14^. Therefore, frail and elderly patients might yield additional benefits from a reduction in surgical invasiveness when compared to a younger population.

Full-endoscopic spine-surgery (FESS) presents an emerging surgical technique that minimizes the invasiveness of spinal surgery^15,16^. Previous reports indicate efficacy and safety of this approach^17,18^. FESS drastically reduces postoperative wound-related complications and virtually eradicates surgery site infection^19^, while also expediting postoperative recovery with significant pain alleviation within the first few days^20^. Additionally, FESS facilitates awake anesthesia which reduces anesthetic complications, more notably in frail populations^21,22^. In doing so, FESS has shown success in minimizing wound-related issues and functional disparities in the postoperative recovery for obese and non-obese patients^23,24^. Thus, FESS enables an instant postoperative mobilization which might yield further benefits in frail patients.

As of today, only limited research analyzing the intersection of frailty and FESS exists. Hence, the aim of this work was to assess the safety profile of FESS in a frail population as well as outlining potential differences in recovery patterns when compared to a non-frail population. For this study, the CCI was utilized as a frailty surrogate.

## Materials and methods

### Data collection

This study was a retrospective analysis of patient data prospectively obtained at one national center. Inclusion criteria encompassed patients aged 18 years and above who had undergone FESS for degenerative pathologies of the lumbar spine (including the thoracolumbar and lumbosacral junction) between March 2014 and May 2023. All surgeries were performed by the same, experienced surgeon. Data was retrieved through the patients’ electronic medical records. FESS, in this context, was defined as the utilization of a uniportal working channel endoscope equipped with a light source, camera, and an irrigation channel. We excluded all patients with traumatic and malignant pathologies, as well as procedures involving a hybrid approach that integrated minimally invasive or open techniques, fusion surgeries, and cervical or thoracic surgeries. All data were obtained pseudonymously in accordance with national law and the declaration of Helsinki. Written and informed consent were obtained from all patients prior to the surgery. Data was obtained following the approval of the Institutional Review Board of the University of Washington (IRB No. 07742). The data that support the findings of this study are not openly available due to reasons of sensitivity and are available from the corresponding author upon reasonable request.

### Outcomes

Demographic parameters encompassed sex, age, race, and Body Mass Index (BMI). The Charlson Comorbidity index was utilized as an indicator for frailty^25^. Furthermore, comorbidities such as hypertension, diabetes, depression, smoking status, chronic heart failure, diabetes, and chronic obstructive pulmonary disease (COPD) were included and analyzed separately. Surgical details covered anatomical location, the number of operated levels, and surgery duration. Surgeries were divided into decompression surgeries for spinal canal stenosis, and discectomies for disc herniations. Hospitalization data and postoperative quality indicators, including the duration of stay, emergency department utilization, clinic readmission, and surgical revision, were also gathered. Patient-reported outcome measures (PROMs) were collected prospectively at any in-person follow-up, which included a Visual Analogue Scale (VAS) for back pain and in the affected extremity, as well as the Oswestry Disability Index as a functional outcome^26^. For analysis, a CCI ≥ 3 as a surrogate for multimorbidity and frailty was used as a cut-off threshold for the frail and non-frail group^25^. The primary study outcomes included length of stay, occurrence of emergency department utilization, clinic readmissions, and surgical revisions within ninety days post-surgery. Additionally, PROMs were compared postoperatively between the frail-group and the non-frail-group at the two-week, three-month, and chronic timepoint. Chronic was defined as at least six months after the initial surgery.

All data acquisition procedures adhered to the Institutional Review Board’s approval. Pseudonymous data collection was conducted in compliance with national law and the Declaration of Helsinki. Written and informed consent was obtained from all patients prior to the surgery.

### Statistical analysis

Statistical analyses were performed using GraphPad Prism (Version 9.5.0; GraphPad Software, Boston, MA). Continuous variables are presented as means ± standard deviations (SD), and categorical variables are expressed as the number of cases with percentages. Groups were compared using Student’s t-test, Mann-Whitney U test, Chi-square, and Fisher’s exact test when appropriate. A significance level of *p* < 0.05 was applied to all analyses. Minimum Clinically Important Difference (MCID) threshold was set at 1.2 points for back pain and 1.6 points for leg pain. An improvement of 30% in the Oswestry Disability Index (ODI) was considered an MCID^27,28^. MCID calculations were performed at the two-week, three-month, and the chronic timepoint. Thereafter, a multivariate regression model for MCID at the given timepoints adjusting for demographic and surgical parameters was constructed. Lastly, propensity-score matching between our cohorts was performed using a “nearest-neighbor”-method package with a small caliper of 0.01 to ensure tight matching. Matches were based on clinically pertinent parameters including patient sex, BMI, number of surgical levels, and the type of surgery (i.e. discectomy or decompression). Univariate analyses were then repeated for this new matched cohort.

## Results

### Patient population

In total, 585 patients met the inclusion criteria; of those, 329 patients were assigned to the control group, whereas 256 patients were assigned to the group with a CCI ≥ 3. After group comparison, patients of the CCI ≥ 3 group exhibited significantly higher age (69.4 ± 10.3 vs. 48.8 ± 13.0; *p* < 0.001; frail group vs. control group in the following), and a higher number of comorbidities (6.2 ± 4.3 vs. 3.03 ± 4.3; *p* < 0.001) when compared to the control group. Patients with a CCI ≥ 3 underwent FESS decompression (*p* < 0.001) and multilevel surgery (*p* < 0.001) more frequently than the control group. Propensity matching yielded satisfactory results for patient matching of 206 matched pairs. Matching successfully eliminated discrepancies between the groups regarding race (*p* > 0.99), operated levels (*p* = 0.87), and surgical approaches (*p* = 0.97). As expected, significant differences regarding age (*p* < 0.01) and number of comorbidities (*p* < 0.01) remained after propensity matching (Table 1).

**Table 1:** Patient demographics with comorbidities before and after propensity matching.

Variable	Unadjusted			Propensity-matched		
	CCI 0–2 (*n* = 329)	CCI ≥ 3 (*n* = 256)	*p*-value	CCI 0–2 *N* = 206	CCI ≥ 3 *N* = 206	
Sex			> 0.99			> 0.99
Male	200 (60.8%)	156 (60.9%)		122 (59.2%)	122 (59.2%)	
Female	129 (39.2%)	100 (39.1%)		84 (40.8%)	84 (40.8%)	
Race			**0.03**			> 0.99
White	296 (90%)	243 (94.9%)		203 (98.5)	203 (98.5)	
Asian	15 (4.6%)	8 (3.1%)		3 (1.5%)	3 (1.5%)	
African American	10 (3%)	2 (0.8%)		0 (0%)	0 (0%)	
Others	8 (2.4%)	3 (1.2%)		0 (0%)	0 (0%)	
BMI	30.1 (6.63)	29.8 (5.6)	0.97	29.8 (6.4)	29.7 (5.3)	0.96
Obese	133 (40.4%)	100 (39.1%)		80 (38.8%)	80 (38.8%)	
Non-obese	196 (59.6%)	156 (60.9%)		126 (61.2%)	126 (61.2%)	
Operated levels			**< 0.01**			0.87
1	275 (83.6%)	173 (67.6%)		165 (80.1%)	165 (80.1%)	
2	51 (15.5%)	74 (28.9%)		40 (19.4%)	40 (19.4%)	
3	2 (0.6%)	7 (2.7%)		1 (0.5%)	1 (0.5%)	
4	1(0.3%)	2 (0.8%)		0 (0%)	0 (0%)	
Surgery			**< 0.01**			0.97
Discectomy	176 (53.5%)	73 (28.5%)		66 (32%)	66 (32%)	
Decompression	153 (46.5%)	183 (71.5%)		140 (68%)	140 (68%)	
Age	48.8 (13.0)	69.4 (10.3)	**< 0.01**	51.2	69.3	**< 0.01**
Comorbidities						
Smoking	79 (24.1%)	39 (15.2%)	**0.01**	52 (25%)	29 (14,1%)	**0.71**
COPD	4 (1.2%)	13 (5.1%)	**0.01**	3 (1,4%)	12 (5,8%)	**0.01**
Hypertension	102 (31.0%)	180 (70.3%)	**< 0.01**	69 (33.2%)	139 (67.5%)	**< 0.01**
Depression	87 (26.4%)	48 (18.8%)	**0.03**	51 (24.5%)	40 (19.4%)	**0.01**
CHF	4 (1.2%)	13 (5.1%)	**0.01**	1 (0.5%)	8 (3.9%)	**0.02**
Diabetes	34 (10.3%)	76 (29.7%)	**< 0.01**	12 (5.8%)	58 (28.2%)	**< 0.01**
No. comorbidities	3.03 (2.87)	6.2 (4.3)	**< 0.01**	3.21	6.02	**< 0.01**

Cohorts were matched for sex, race, BMI, operated surgical levels, and surgery.

Significant values are in bold.

In the initial analysis significant differences showed between the groups regarding intraoperative blood loss (7.81 ± 6.65 vs. 5.71 ± 5.94; *p* < 0.01) and the duration of surgery (168.8 ± 75.3 vs. 143.6 ± 66.3; *p* < 0.01). Those differences did not remain statistically significant after propensity matching. Postoperatively, patients with a CCI ≥ 3 were treated as inpatients significantly more often (*p* < 0.001) and had a significantly higher number of days stayed in the hospital (0.7 ± 1.5 vs. 0.4 ± 1.2; *p* < 0.001), which stayed statistically significant after propensity matching (*p* < 0.01 and *p* = 0.05). All patients were discharged home postoperatively. Regarding post-discharge clinic utilization, no statistically significant differences were found between the groups for any postoperative emergency department utilization (*p* = 0.39), clinic readmission (*p* > 0.99), and surgical revision (*p* = 0.514). However, after propensity matching, significant differences showed regarding surgical revisions for those patients with CCI ≥ 3 (1.0% vs. 2.4%; *p* < 0.01) (Table 2).

**Table 2:** Operative details and post-surgical clinic utilization.

Variable	Unadjusted			Propensity-matched		
	CCI 0–2 (*n* = 329)	CCI ≥ 3 (*n* = 256)	*p*-value	CCI 0–2 *N* = 206	CCI ≥ 3 *N* = 206	*p*-value
Previous surgery	118 (35.9%)	88 (34.4%)	0.728	80 (38.8%)	75 (36.4%)	0.68
Duration (min)	143.6 (66.3)	168.8 (75.3)	**< 0.01**	150.7 (67.0)	152.5 (61.0)	0.78
Est. blood loss ml/level	5.71 (5.94)	7.81 (6.65)	**< 0.01**	6.9 (6.2)	7.64 (6.9)	0.27
Inpatient	70 (21.3%)	114 (44.5%)	**< 0.01**	43 (20.9%)	81 (39.2%)	**< 0.01**
Duration of stay	0.4 (1.2)	0.7 (1.5)	**< 0.01**	0.4 (1.3)	0.6 (1.1)	**0.05**
Emergency department utilization, 100d	56 (16.8%)	51 (19.3%)	0.39	43 (18.9%)	36 (14.6%)	0.29
Clinic readmission	9 (2.7%)	7 (2.7%)	> 0.999	4 (1.9%)	7 (2.9%)	0.25
Surgical revision	4 (1.2%)	5 (2%)	0.514	2 (1.0%)	5 (2.4%)	**< 0.01**

Significant values are in bold.

### Functional outcomes

**Fig. 1:**
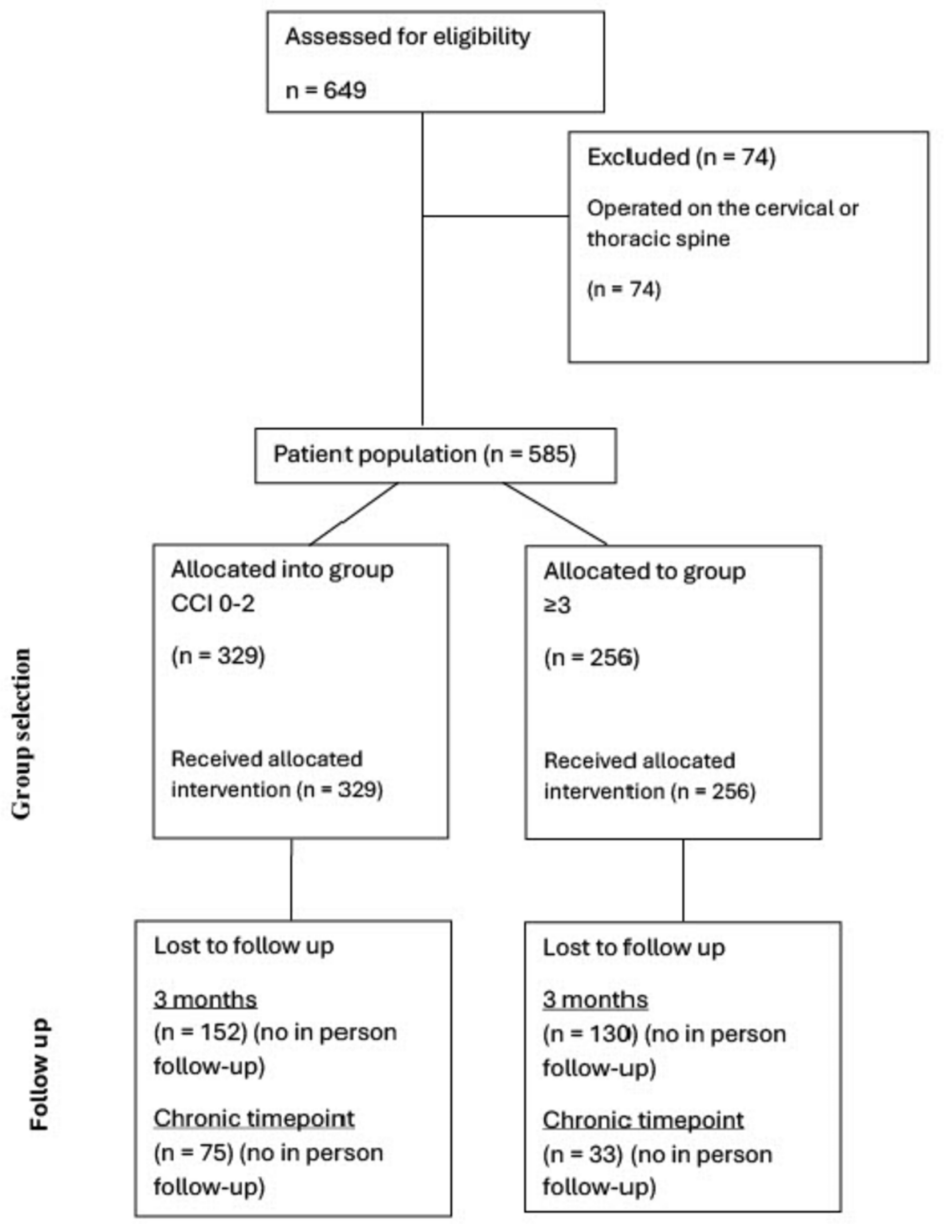
Flow-chart of the patient follow-up for functional outcomes after undergoing full-endoscopic surgery after group selection, at 3-months after the surgery and at the chronic timepoint. Data was available for unplanned clinic utilization and postoperative complications in all patients.

In total, 177 patients from the control group and 114 from the intervention group reported functional outcomes 3 months after the surgery. At the chronic timepoint 102 patients reported functional outcomes for the control group and 73 in the group with CCI ≥ 3 (Fig. 1). At baseline, no statistically significant differences were observable between the groups for back pain, leg pain, and ODI. Patients with CCI ≥ 3 patients showed a slower functional recovery as expressed by a significantly smaller ODI improvement at the two-week timepoint (∆3.52 ± 10.5 vs. ∆5.37 ± 10.2, *p* = 0.044). At the chronic timepoint, patients with CCI ≥ 3 reported significantly smaller benefits in ODI improvement when compared to the control group (∆5.49 ± 10.7 vs. ∆8.11 ± 10.6; *p* = 0.047) which translated into a significantly smaller probability of achieving an MCID (*p* = 0.018) in patients with CCI ≥ 3. This remained statistically significant after propensity matching (*p* = 0.02). No statistically significant differences were found for pain alleviation at any timepoint (Table 3).

**Table 3:** Patient reported outcomes.

Variable	Unadjusted			Propensity-matched		
	CCI 0–2 (*n* = 329)	CCI ≥ 3 (*n* = 256)	*p*-value	CCI 0–2 *N* = 206	CCI ≥ 3 *N* = 206	*p*-value
	*n* = 177	*n* = 126		*n* = 114	*n* = 100	
3 months						
∆ VAS back	2.18 (3.1)	2.83 (3.23)	0.922	2.53 (3.11)	2.09 (3.22)	0.32
MCID back	100 (58.1%)	62 (49.2%)	0.158	67 (59.3%)	43 (44.8%)	**0.04**
∆ VAS leg	3.35 (3.28)	2.73 (3.33)	0.12	3.39 (3.26)	2.73 (3.51)	0.16
MCID leg	124 (70.1%)	69 (56.6%)	**0.02**	83 (72.8%)	52 (55.9%)	0.01
∆ ODI	9.72 (10.2)	8.68 (10.3)	0.198	8.97 (9.95)	8.56 (10.01)	**0.01**
MCID ODI	104 (61.2%)	73 (57%)	0.477	64 (56.6%)	58 (58.0%)	0.89

	*n* = 102	*n* = 93		*n* = 73	*n* = 77	
Chronic						
∆ VAS back	1.42 (2.8)	1.61 (3.13)	0.747	1.6 (2.9)	1.29 (3.01)	0.53
MCID back	40 (39.2%)	39 (41.9%)	0.771	31 (42.5%)	28 (37.3%)	0.61
∆ VAS leg	1.94 (3.42)	2.4 (4.15)	0.57	1.72 (3.35)	2.41 (4.1)	0.27
MCID leg	47 (47%)	42 (48.3%)	0.884	30 (43.5%)	35 (49.3%)	0.5
∆ ODI	8.11 (10.6)	5.49 (10.7)	**0.047**	7.71 (10.63)	4.86 (11.03)	0.12
MCID ODI	47 (50.5%)	31 (32.6%)	**0.02**	32 (49.2%)	21 (27.3%)	**0.01**
Time to chronic (days)	441 (319)	477 (326)	0.6	416.9 (286.3)	471.9 (330.1)	0.24

Delta refers a change in the respective reported outcome when compared to baseline. A positive delta indicates an improvement, a negative delta indicates an aggravation.

Significant values are in bold.

### Multivariate analysis

Multivariate analysis revealed a CCI ≥ 3 to be significantly correlated with reported MCID for ODI (OR 0.5, *p* = 0.023, 95% CI 0.2 to 0.9) at the chronic timepoint. Likewise, a preoperatively diagnosed depression showed significant correlation with reported MCID for back pain (OR 0.4, *p* = 0.25, 95% CI 0.2 to 0.9), and female sex with reported MCID for leg pain (OR 0.4, *p* = 0.008, 95% CI 0.2 to 0.8) (Table 4).

**Table 4:** Multivariate logistic regression for MCIDs at the chronic time point (> 6 months).

Parameter	MCID back pain, chronic			MCID leg pain, chronic			MCID ODI, chronic		
	OR	*p*-value	95% CI	OR	*p*-value	95% CI	OR	*p*-value	95% CI
Sex (female)	0.5	0.058	0.3 to 1.0	**0.4**	**0.008**	**0.2 to 0.8**	1.2	0.593	0.6 to 2.3
Race	0.9	0.949	0.6 to 1.5	0.7	0.137	0.4 to 1.1	1.1	0.598	0.7 to 1.9
Obese	1.1	0.801	0.6 to 2.0	1.0	0.934	0.5 to 1.9	1.1	0.827	0.6 to 2.1
CCI ≥ 3	1.1	0.867	0.6 to 2.0	0.7	0.386	0.4 to 1.4	**0.5**	**0.023**	**0.2 to 0.9**
Depression	**0.4**	**0.025**	**0.2 to 0.9**	1.3	0.422	0.7 to 2.8	1.5	0.329	0.7 to 3.1
Operated levels	0.9	0.872	0.4 to 2.1	**0.4**	**0.036**	**0.2 to 0.9**	0.7	0.364	0.3 to 1.5
Discectomy	1.4	0.365	0.7 to 2.8	0.6	0.143	0.3 to 1.2	0.5	0.064	0.3 to 1.0
Inpatient	1.0	0.988	0.5 to 2.0	0.7	0.251	0.3 to 1.3	1.8	0.117	0.9 to 3.8
Surgery duration	1.0	0.066	0.9 to 1.0	1.0	0.596	0.9 to 1.0	**1.0**	**0.033**	**0.9 to 1.0**

Significant values are in bold.

## Discussion

The presented study underlines the feasibility and safety of FESS in patients with relevant CCI scores. Furthermore, the results indicate disparities in reported benefits of the surgical procedure. While pain alleviation following FESS was similar between patients with CCI ≥ 3 and the control group, patients with CCI ≥ 3 appear to yield lower functional benefits at chronic postoperative timepoints.

Demographic aging will inevitably change the patient population undergoing elective spine surgery. Consequently, it is imperative that contemporary spine surgery establishes safe and effective surgical procedures for spinal pathologies in multimorbid patients while addressing their increased risk of adverse events^8–12^. Furthermore, all surgical considerations need to take resource scarcity in global healthcare systems into account^1^. FESS has established itself as a minimally invasive surgical technique that can safely be performed in an outpatient setting^29^. Regarding the intersection of multimorbidity, age, and FESS, only a little body of research exists that has shown feasibility and efficacy in an aged population^30^. Compared to open procedures, FESS has been associated with a shorter length of hospital stay and lower non-home discharge in patients with comorbidities^31^. However, there is a lack of research comparing those patient populations undergoing FESS. Our study corroborates the previously reported feasibility, safety, and efficacy of FESS in patients with comorbidities and the elderly. The cutoff value of a CCI ≥ 3 is substantiated by the patient populations’ demographics reflected by a higher age as well as age-associated comorbidities. Most importantly, no differences in postoperative safety measures and quality indicators were observable between the cohorts. Emergency department utilization, clinic readmission, and surgical revisions as essential point-of-care measures were similar between groups^32^. When compared to other surgical approaches, these findings add potential advantages of a minimized surgical invasiveness since patients with medical conditions have shown higher readmission rates, perioperative complications, and discharge deposition^6,12^. In the presented cohort, all patients were discharged home.

Due to those patients with CCI ≥ 3 being among an aged population, naturally, this cohort required inpatient treatment more often and consequentially had a higher length of stay which is supported by the literature in traditional spine surgery^11^. However, after the propensity matching, a significant difference showed regarding the surgical revision rates between the groups.

In addition, pathophysiological differences between the two groups were observed. Patients with CCI ≥ 3 more frequently underwent decompression for multilevel pathologies while the control population underwent single level surgery due to spinal stenosis and disc herniation at similar rates^33–35^. Interestingly, patients with CCI ≥ 3 who seemingly underwent more complex multilevel procedures reported similar postoperative pain improvements at any timepoint. However, patients with CCI ≥ 3 reported a significantly lower ODI improvement and MCID at the chronic timepoint. This finding is supported by studies indicating smaller ODI improvements for frail patients undergoing lumbar fusion and by our multivariate analysis^36,37^. While radiographic parameters were not included in the analysis, one potential explanation for this could be a more complex underlying pathology in the aging spine. Younger patients typically present with single level disc herniations, as evidenced by the overall higher number of discectomies in the control cohort. As opposed to elderly, often times presenting with multiple pathologies, including degenerative scoliosis and sagittal malalignment, which is especially prevalent in a frail population^38,39^. In spite of feasibility studies and case series of FESS fusion surgeries, a standardized surgical approach for the correction of sagittal malalignments remains a limitation of FESS^40,41^. For our cohort, some of the elderly patients might have yielded additional benefits from a spinal realignment procedure.

Our reported results are essential for preoperative patient counselling as they elucidate different recovery patterns. While multimorbid patients can expect significant improvements in back and leg pain after the surgery, the improvement of low-back disability is limited by their age-associated overall condition.

The multivariate analysis indicated a significant correlation between preoperative depression and the achievement of an MCID for back pain at the chronic timepoint. A high prevalence of depression in patients with chronic back pain has been reported^42^. Notably, depressed patients appear to yield smaller benefits from spine surgery than their non-depressed counterparts^43^. This finding is supported by several studies identifying preoperative depression as a predictor for lower patient satisfaction and higher adverse events surrounding spine surgery^43–45^. The intersection of backpain is multifaceted with implications for both preoperative counselling and postoperative rehabilitation. While depressed patients report overall improvements for both back pain, and depression scores, implications of the depressive disorder need to be discussed with the patients preoperatively^46^. Moreover, female sex and more operated levels had a significant correlation with reported MCID for leg pain in the multivariate analysis. While complexity of the surgery and multilevel pathology appear to be plausible explanations for a lower MCID in this population, there is an ongoing debate about sex affecting functional outcomes after spine surgery and its’ clinical significance^47–49^. While the significance of sex disparities in the presented study should be interpreted with caution, sex differences in perceived postoperative pain improvement is essential for comprehensive patient counselling.

FESS demonstrates a minimally invasive approach that benefits frail patients with regard to the populations increased susceptibility to surgery complications previously described. FESS in frail patients remains a safe and efficient method to sufficiently address spinal pathologies while substantially reducing surgical invasiveness.

### Limitations

The authors acknowledge that this work has limitations. The retrospective analysis of prospectively collected data implies selection bias. Furthermore, data assessments were conducted at the in-person follow-ups resulting in relevant loss-to follow-up for reported functional outcomes. This is especially relevant as many patients don’t attend their 3-month or chronic follow-ups which affects completeness of the data, presumably mainly for those patients who are doing well and thus underestimating the actual postoperative pain and functional improvements. In our cohort, only approximately half of the patients had an follow-up after 3 months, and only one third of the patients after 6 months, which presents a key limitation for the functional outcomes of our patients at the given timepoints. This could be prevented with modern tools (e.g. smartphone applications) that enable asynchronous patient feedback to generate larger cohorts regarding secondary outcomes following FESS^50^. The single-center, single-surgeon design of the study limits the generalizability of the results. Lastly, the grouping presents a limitation itself as numerous assessments for frailty and multimorbidity exist. For this study, the CCI was chosen as it includes a broad number of comorbidities as well as age as an important factor for preoperative patient counselling and selection. However, other frailty assessments or surrogates might have yielded different results.

## Conclusion

FESS is feasible and safe in elder patients with comorbidities with an overall low complication rate regarding emergency revision surgery and postoperative hospital readmission. However, multimorbidity should always elicit apprehension in an ambulatory setting as a relevant number of those patients require postoperative inpatient care. These results provide guidance regarding preoperative patient counselling. While patients with comorbidities can expect similar postoperative pain reduction following FESS, they might yield smaller functional benefits than their healthier and younger counterparts.

## Data Availability

The data that support the findings of this study are not openly available due to reasons of sensitivity and are available from the corresponding author upon reasonable request.
